# Ultrasound-Assisted Extraction May Not Be a Better Alternative Approach than Conventional Boiling for Extracting Polysaccharides from Herbal Medicines

**DOI:** 10.3390/molecules21111569

**Published:** 2016-11-18

**Authors:** Ka-Man Yip, Jun Xu, Wing-Sum Tong, Shan-Shan Zhou, Tao Yi, Zhong-Zhen Zhao, Hu-Biao Chen

**Affiliations:** School of Chinese Medicine, Hong Kong Baptist University, Hong Kong, China; 11015551@life.hkbu.edu.hk (K.-M.Y.); 12017817@life.hkbu.edu.hk (W.-S.T.); 15485404@life.hkbu.edu.hk (S.-S.Z.); yitao@hkbu.edu.hk (T.Y.); zzzhao@hkbu.edu.hk (Z.-Z.Z.)

**Keywords:** ultrasound-assisted extraction, extraction conditions, structural features, polysaccharides, herbal medicines, degradation

## Abstract

In clinical practice polysaccharides from herbal medicines are conventionally prepared by boiling water extraction (BWE), while ultrasound-assisted extraction (UAE) has often been used instead employed in laboratory research due to its strong extraction ability and efficiency. However, if and how the polysaccharides obtained by UAE and BWE are comparable, and hence whether the UAE-based research is instructive for the actual usage of herbal polysaccharides still requires further evaluation. To address this issue, here we chemically analyzed and compared the UAE- and BWE-obtained polysaccharides from three herbal medicines, i.e., Ginseng Radix, Astragali Radix and Dendrobii Officinalis Caulis. Then, the spike recovery of two series of standard dextran and pullulan by UAE and BWE was tested. The results showed that the polysaccharides from the herbal medicines by UAE were quantitatively and qualitatively different with those by BWE. The powerful extraction ability and polysaccharide degradation caused by ultrasound collectively contributed to these differences. It was then revealed that not only the UAE conditions but also the polysaccharide structures could affect the extraction ability and polysaccharide degradation. Given these, we highly recommended that the effects of UAE on polysaccharides from herbal medicines should be first carefully considered before employing it in relevant chemical and pharmacological analysis.

## 1. Introduction

Polysaccharides have been becoming progressively appreciated as some of the most important kinds of chemical ingredients in herbal medicines due to their various medicinal values, such as anticancer [1], immune regulation [2], hyperglycemic [3,4] and prebiotic-like effects [5]. Intensive research interests have been therefore focused on polysaccharides in herbal medicines. Nevertheless, nowadays natural polysaccharide research still suffers from multidisciplinary methodological bottlenecks: structural elucidation [6], quality control [7], in vivo detection [8] and in vivo molecular target exploration [9,10,11]. However, as the first step to tackle these challenges, sample extraction is equally important and still needs more consideration [12].

For thousands of years, herbal medicines were generally prepared by repeated water boiling. For example, in traditional Chinese medicine (TCM), decoctions (boiled water extracts) are the most commonly used medication form for disease treatment [13,14]. That is to say, in long-term clinical practice, polysaccharides obtained by boiling water extraction (BWE) are the effective substances (taken forms) of the whole polysaccharides originally-occurring in the herbal medicines. However, recently ultrasound-assisted extraction (UAE) using bath or probe-based systems has often been employed instead of BWE for extracting polysaccharides from herbal medicines for further chemical or pharmacological testing in relevant laboratory research [15,16,17,18,19,20,21,22]. The multifaceted advantages of UAE over conventional BWE for natural product/food extraction have been reported, in which stronger extraction ability and efficiency are typically highlighted [23,24]. However, it has been demonstrated that UAE could degrade certain natural polysaccharides with different structural properties, namely pectin, carrageenan, chitosan and starch, thereby changing their chemical, physical and biological properties, and multiple UAE parameters (e.g., ultrasonic power, extraction time and temperature) could substantially affect such degradation [25,26,27].

Herein we pose the questions, from a holistic perspective, of whether and how UAE impacts the various polysaccharides in herbal medicines, and thereby extracts quantitatively and qualitatively different total polysaccharides compared to conventional BWE. The issues above are significant to evaluate whether the UAE-related research is instructive for the actual usage of herbal polysaccharides. Although certain differences between total polysaccharides from same herbal medicine by UAE and BWE have been indeed found in several cases [20,28,29,30,31], these studies focused on only individual samples, from which overall findings and conclusions were difficult to obtain. Furthermore, most of them did not adequately consider the UAE parameters as well as the structural properties of herbal polysaccharides, both of which could be decisive for the extraction outcomes. For example, some reports have employed UAE without optimizing the conditions [28,29], while others only compared the extraction yields [30,31].

Thus a more systemic and further investigation was performed in this study. Three frequently-used herbal medicines, namely Ginseng Radix *et* Rhizome (GR) (the root and rhizome of *Panax ginseng*), Astragali Radix (AR) (the root of *Astragalus membranaceus*), and Dendrobii Officinalis Caulis (DO) (the stem of *Dendrobium officinale*) were selected as study models considering their dominant polysaccharide components [32,33,34]. We chemically investigated and compared the polysaccharides obtained by UAE and BWE from the three herbal medicines by phenol-sulphuric acid analysis and high performance gel permeation chromatography coupled with charged aerosol detector (HPGPC-CAD) analysis. To achieve the optimal extraction conditions, UAE was examined by response surface methodology (RSM), for which was also examined to see how UAE conditions impact the herbal polysaccharides obtained; while BWE was optimized by repeated boiling. Afterwards, two series of natural product-derived standard glucans, namely dextran and pullulan, with different structural features in polymerization degrees, types of sugar chain and glucosidic linkage were extracted by the optimized UAE and BWE conditions to further investigate how the structural properties of polysaccharides affected their recovery.

## 2. Results and Discussion

### 2.1. Optimization of Sample Preparation

In order to equally compare the UAE- and BWE-obtained polysaccharides under the maximum extraction ability (polysaccharide yields) of the two methods, the conditional parameters for UAE and BWE were optimized by RSM with BBD and repeated boiling, respectively.

#### 2.1.1. UAE

RSM with BBD is an effective statistical technique for optimizing complex extraction processes involving multiple conditional parameters [35]. By a sequence of designed experiments, it generates a quadratic model to explore the relationships between several explanatory variables and one or more response variables, which are difficult to be elucidated by conventional optimization method, e.g., single factor analysis. RSM with BBD has been widely adopted to optimize the UAE conditions for natural polysaccharides [16,18,19,20,21,22,31]. Therefore it was employed here to acquire the optimal conditions for UAE of GR, AR and DO polysaccharides, and the procedure was carried out as follows.

##### Statistical Analysis and Model Fitting

The corresponding extraction yields of 15 runs using BBD design for the three natural samples were presented in Table 1.

The complete quadratic equation describing the relationship between the independent variables and response variable of each herbal medicine was given by the following equations:

(1) $$\begin{array}{cc} \mathrm{GR}: & Y=46.64-2.69X_{1}+0.21X_{2}+0.20X_{3}-0.70X_{1}X_{2}+0.14X_{1}X_{3}-2.06X_{2}X_{3}-12.46{X_{1}}^{2}-0.41{X_{2}}^{2}-3.73{X_{3}}^{2} \end{array}$$

(2) $$\begin{array}{cc} \mathrm{AR}: & Y=4.03+1.88X_{1}+0.44X_{2}+0.13X_{3}+0.30X_{1}X_{2}-0.095X_{1}X_{3}+0.075X_{2}X_{3}-1.46{X_{1}}^{2}+0.05{X_{2}}^{2}-0.057{X_{3}}^{2} \end{array}$$

(3) $$\begin{array}{cc} \mathrm{DO}: & Y=41.34+2.92X_{1}-0.86X_{2}+1.89X_{3}-0.038X_{1}X_{2}-3.84X_{1}X_{3}-0.72X_{2}X_{3}-6.49{X_{1}}^{2}-1.29{X_{2}}^{2}-0.96{X_{3}}^{2} \end{array}$$

The analysis of variance (ANOVA) of the regression model of each sample analyzed by Design-Expert 8.0 is presented in Table 2.

The large model *F*-value and small *p*-value (*p* < 0.05) indicated that the coefficients were statistically significant. The determination coefficients were 0.9696 for GR, 0.9912 for AR and 0.9480 for DO, which suggested that 96.96%, 99.12% and 94.80% of the total variations of each model could be accounted for by the model. The adjusted determination coefficient was also in reasonable agreement with the predicted determination coefficient of each sample as the difference between the two coefficients was within 0.2, which suggested the predicted and observed values of the three models were in high correlation. A relatively low coefficient variation value in the three regression models (C.V. = 5.50% for GR; C.V. = 8.03% for AR; C.V. = 5.13% for DO) revealed a good precision and reliability of the model to predict experimental results. The significance of the regression model was also determined by the lack of fit test. As the lack of fit test of the three herbal samples resulted in a large *p*-value (*p* = 0.6330 for GR; *p* = 0.3538 for AR; *p* = 0.9407 for DO), this implied that the lack of fit was not significant relative to the pure error, which confirmed the goodness-of-fit and suitability of the model for prediction of the response values under any combination of the independent variables.

##### Verification of Predictive Model

From the statistical analysis, the predicted optimal extraction conditions for each herbal medicine are summarized in Table 3. To verify the suitability of the model equation for prediction, triplicate confirmatory experiments under the optimal condition with slight modification were carried out. The average yields of polysaccharide of the three herbal samples were very close to the predicted values, which suggested the model was suitable for the optimization of extraction condition. The extraction efficiency by UAE was indeed improved by using a lower temperature and shortened extraction time as compared with BWE. The polysaccharides from different sources required different optimal extraction conditions, and interestingly that lower polysaccharide yield did not seem to warrant easier extraction.

For example, the polysaccharide yield of AR (5.43%) was far less than that of DO (42.78%), but the UAE conditions was much harsher for the polysaccharide preparation of AR than DO, i.e., higher temperature (82.19 °C for AR; 69.31 °C for DO) and longer extraction time (72 min for AR; 44 min for DO). This phenomenon reflects well the diversity of polysaccharides from herbal medicines.

#### 2.1.2. BWE

In order to simulate the traditional clinical practice of Chinese medicines, the sample powder of each herbal medicine was refluxed repeatedly at 100 °C (1 h each time) until no sugar was detected in the subsequent extraction by phenol-sulphuric acid analysis. With two extractions, the polysaccharides in GR, AR and DO could be completely extracted.

### 2.2. Quantitative Comparison of Polysaccharides in Herbal Medicines by UAE and BWE

**Figure 1:**
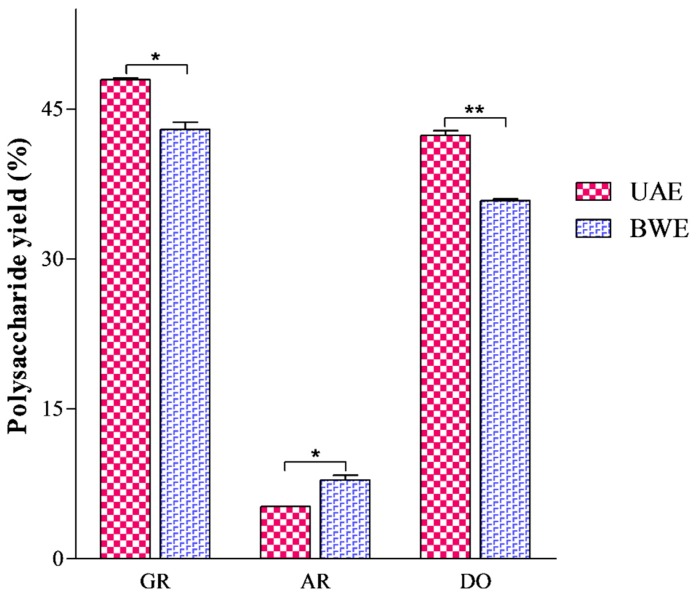
Quantitative comparison of polysaccharides by UAE and BWE * *p* < 0.05; ** *p* < 0.01.

To reveal the differences between UAE and BWE, we first quantitatively compared the polysaccharides extracted from GR, AR and DO by these two methods under their respective optimal conditions using phenol-sulphuric acid analysis. As shown in Figure 1, the extraction yields of polysaccharides by UAE and BWE were significantly different for each sample (*p* < 0.05 for GR and AR; or *p* < 0.01 for DO). To be specific, UAE extracted more polysaccharides from GR and DO but less from AR compared with BWE (GR: 47.95% by UAE and 42.99% by BWE; DO: 42.37% by UAE and 35.86% by BWE; AR: 5.24% by UAE and 7.88% by BWE).

Previous studies always asserted higher yields by UAE than BWE for polysaccharide extraction from herbal medicines [30,31]. However, our study demonstrated that UAE can potentially produce lower polysaccharide yield than BWE, just as in the case of AR.

### 2.3. Qualitative Comparison of Polysaccharides in Herbal Medicines by UAE and BWE

**Figure 2:**
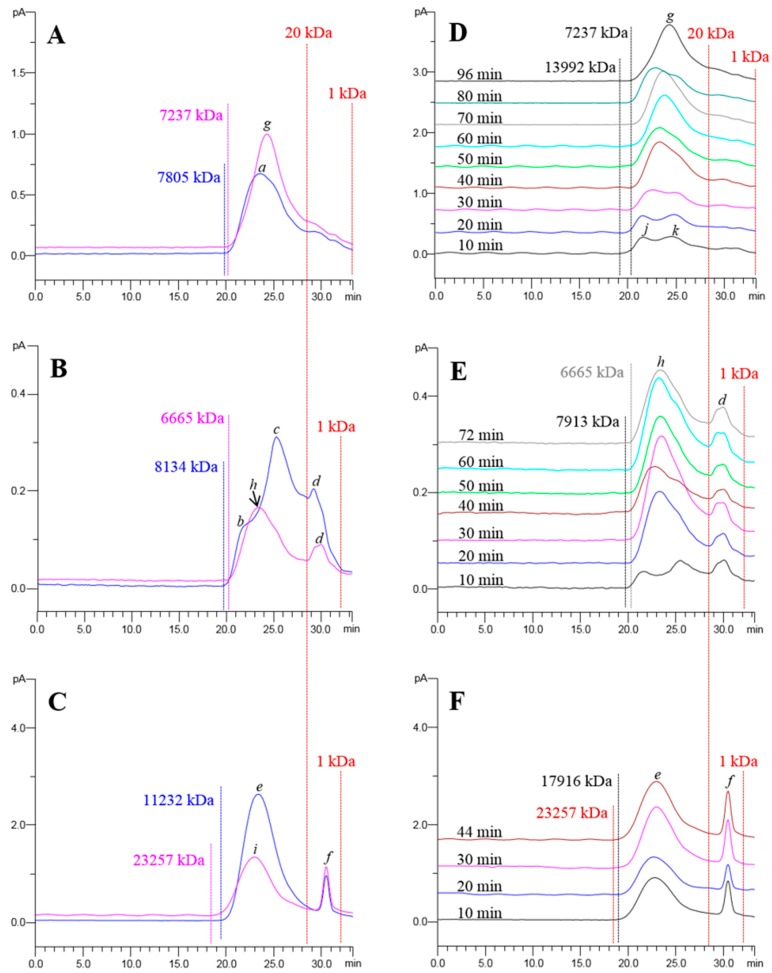
HPGPC-CAD chromatograms of polysaccharides by UAE and BWE (**A**–**C**, –– UAE; –– BWE) and dynamically-extracted polysaccharides by UAE (**D**–**F**) from GR (**A**,**D**), AR (**B**,**E**) and DO (**C**,**F**).

The quantitative analysis preliminarily indicated that the herbal polysaccharides extracted by UAE and BWE could be different. Therefore we further qualitatively compared the polysaccharides from GR, AR and DO obtained by UAE and BWE using HPGPC-CAD. The HPGPC-CAD chromatograms of GR, AR and DO polysaccharides by UAE and BWE were shown in Figure 2A–C.

The peaks showed no obvious absorbance under UV 260 nm and 280 nm (data not shown), suggesting the absence of abundant free and conjugated nucleic acids or proteins and therefore no significant interference with polysaccharide analysis under the conditions used. It was obviously seen that the polysaccharide profiles from GR, AR and DO by conventional BWE differed completely in both molecular weight distribution and peak patterns. Calculated by the established molecular weight-retention time calibration curve (*y* = −0.2982*x* + 9.8266 with R^2^ = 0.9873), BWE-obtained polysaccharides from GR and AR possessed similar molecular weight distributions of 1.37 kDa to 7805.85 kDa and 1.55 kDa to 8134.15 kDa, respectively, while those from DO exhibited a wider range from 1.27 kDa to 11,232.23 kDa. In addition, the chromatograms of GR, AR and DO polysaccharides by BWE gave dominant peaks at different retention times with distinct molecular weights: peak **a** (23.55 min, 635.47 kDa) for GR; peaks **b** (21.49 min, 2612.70 kDa), **c** (25.28 min, 193.74 kDa), **d** (29.25 min, 12.73 kDa) for AR, peaks **e** (23.33 min, 742.65 kDa) and **f** (30.51 min, 5.37 kDa) for DO. The chromatograms further demonstrated the variability of herbal medicinal polysaccharides. With respect to UAE, we were amazed to find that the UAE-obtained polysaccharides (Figure 2A–C) showed significantly different qualitative characteristics compared to the BWE-obtained polysaccharides. For example, UAE narrowed the molecular weight distributions of GR (1.37 kDa to 7237.99 kDa) and AR (1.55 kDa to 6665.52 kDa) polysaccharides compared with BWE. Moreover, peak **a** in GR moved rearward by UAE, producing a higher peak **g** (23.55 min to 24.30 min, 635.47 kDa to 379.70 kDa); peaks **b** and **c** in AR disappeared, instead peak **h** occurred in the same area. These phenomena suggested that UAE likely resulted in the degradation of certain polysaccharides in GR and AR. Conversely, the maximum molecular weight of BWE-obtained DO polysaccharides was greatly increased from 11,232.23 kDa to 23,257.23 kDa by UAE. Accordingly, the dominant peak **e** shifted forward compared with peak **i** (23.33 min to 22.95 min, 742.65 kDa to 963.40 kDa). The broadened molecular weight range for DO polysaccharides by UAE might be owing to the stronger extraction ability of UAE over BWE. Besides, we also noticed that the effects of UAE on polysaccharides from herbal medicines varied with molecular weights, for which polysaccharides with higher molecular weights appeared to be more susceptible. As shown in Figure 2A–C, the molecular weight distribution and peak pattern of polysaccharides larger than 20 kDa in the samples were drastically changed by UAE, whereas the sub-20 kDa peaks (**d** and **f**) kept relatively constant between UAE and BWE. Previous studies revealed that natural polysaccharides, e.g., pectin [36] and chitosan [37], with higher molecular weight could be more easily depolymerized. Here the similar rule was also observed in herbal medicines. Moreover, the degradation might mainly occur at the center rather than the terminal of sugar chains [25], leading to the great changes in the up-20 kDa area (e.g., peak shift) but the minor changes in the sub-20 kDa area. Various characteristic heteropolysaccharides with molecular weights more than 20 kDa have been found in GR [38], AR [39], DO [40]. UAE might degrade these polysaccharides and thereby change their bioactivities, which deserves further investigation. In a word, the powerful energy input of ultrasonic-driven extraction and polysaccharide degradation might collectively contribute to the differences between UAE-obtained polysaccharides and those obtained by BWE.

### 2.4. Effects of Extraction Conditions on Polysaccharide Recovery by UAE

To further elucidate how the optimal UAE conditions affected the extraction ability and polysaccharide degradation during the whole extraction process, the extract of each sample by UAE was dynamically monitored by HPGPC-CAD for every ten minutes until the optimal extraction time was reached. The chromatograms are summarized in Figure 2D–F. We discovered that sub-20 kDa polysaccharides (e.g., peaks **d** and **f**) in the three samples were easily extracted even by the first 10 min extraction and were not significantly changed by the subsequent extraction, whereas polysaccharides above 20 kDa required longer duration extraction and underwent irregular variation with the full-time extractions. In the dynamic extraction of GR polysaccharides (Figure 2D), the molecular weight distribution of extracted polysaccharides was gradually narrowed from a range of 1.37–13,992.33 kDa to 1.37–7237.99 kDa along with the increase of extraction time, indicating that the ultrasonic-induced degradation of polysaccharides continually occurred during the whole extraction. Specifically, the first 10 min extraction generated two peaks (**j** and **k**) above 20 kDa. The increased extraction yield thereafter merged the two peaks into the dominant peak **g** after 40 min extraction. However, the peak **g** was not persistently increased by the following extraction, but remarkably decreased twice at 50 min and 80 min, almost resulting in the re-emergence of the peaks **j** and **k**. This suggested that substantial degradation of polysaccharides by UAE happened at or before these two time points. The peak **g** accordingly shifted backward from 23.26 min (778.15 kDa) by 40 min extraction to 24.30 min (379.70 kDa) by 96 min extraction. The dynamic extraction of AR polysaccharides exhibited a similar tendency as that of GR polysaccharides, i.e., the narrowing down of the molecular weight distribution (from the range of 1.55–7913.78 kDa to 1.55–6665.52 kDa), the retrograding of dominant peak **h** (from 23.30 min, 754.48 kDa to 23.34 min, 736.56 kDa) as well as the substantial decrease of the dominant peak **h** (at or before 40 min and 72 min, respectively, Figure 2E). Nevertheless, UAE widened the molecular weight distribution of DO polysaccharides from 1.27–17,916.04 kDa at 10 min to 1.27–23,257.23 kDa at 44 min with the constantly heightened dominant peak **e**. This suggested that the UAE ability for DO polysaccharide extraction was elevated in pace with the increased extraction time. Even so UAE still possibly broke down the DO polysaccharides, evidenced by the slightly rearward peak **e** (from 22.81 min, 1056.97 kDa to 22.95 min, 963.40 kDa) (Figure 2F). The above facts demonstrated that the effects of the optimal UAE conditions on extraction ability and polysaccharide gradation could gradually or suddenly occurred, varying in different samples, collectively resulting in the final extraction recovery. Besides, it also confirmed our previous speculations that polysaccharides in herbal medicines with higher molecular weight were more susceptible to UAE-induced degradation.

**Figure 3:**
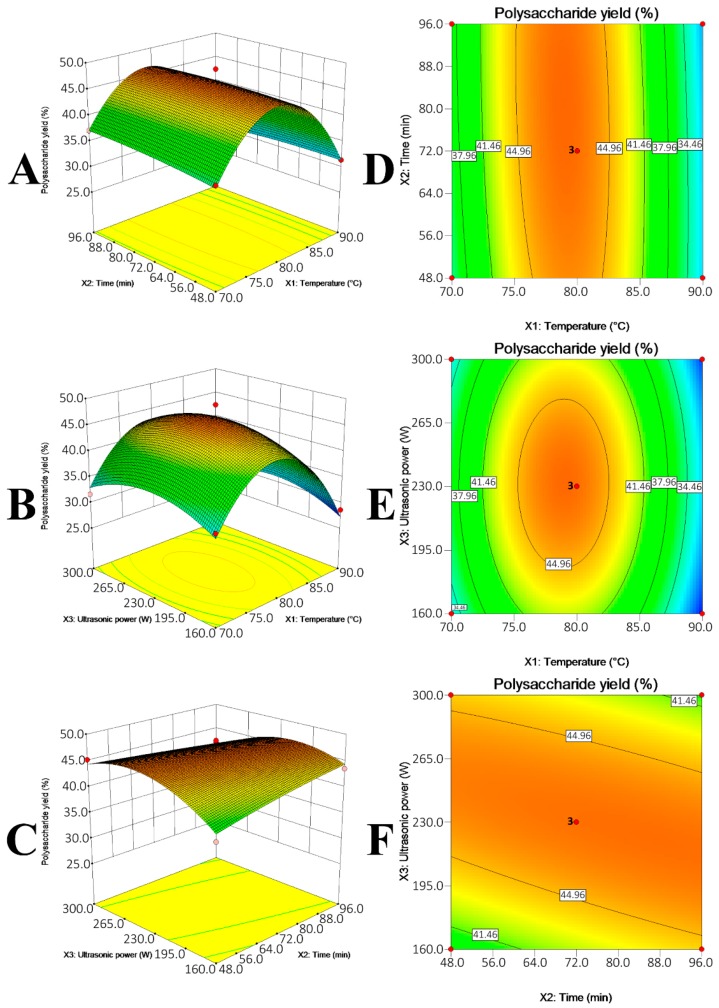
Response surface plot (**A**–**C**) and contour plots (**D**–**F**) showing the effects of temperature (*X*_1_), extraction time (*X*_2_) and ultrasonic power (*X*_3_) on the polysaccharide yield (*Y*) of GR.

**Figure 4:**
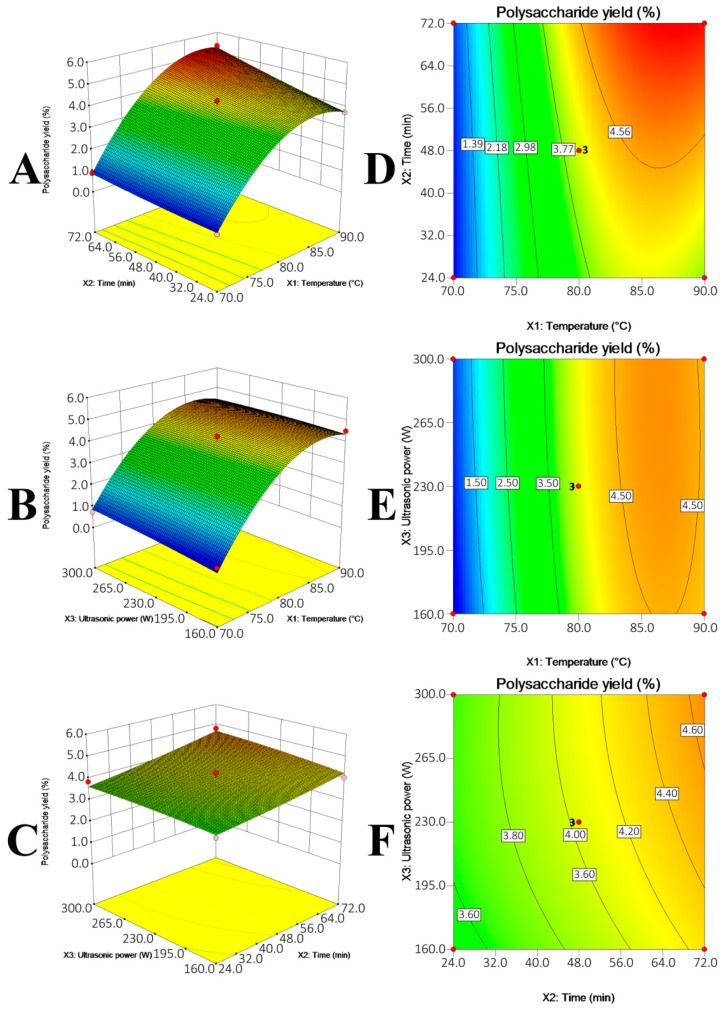
Response surface plot (**A**–**C**) and contour plots (**D**–**F**) showing the effects of temperature (*X*_1_), extraction time (*X*_2_) and ultrasonic power (*X*_3_) on the polysaccharide yield (*Y*) of AR.

**Figure 5:**
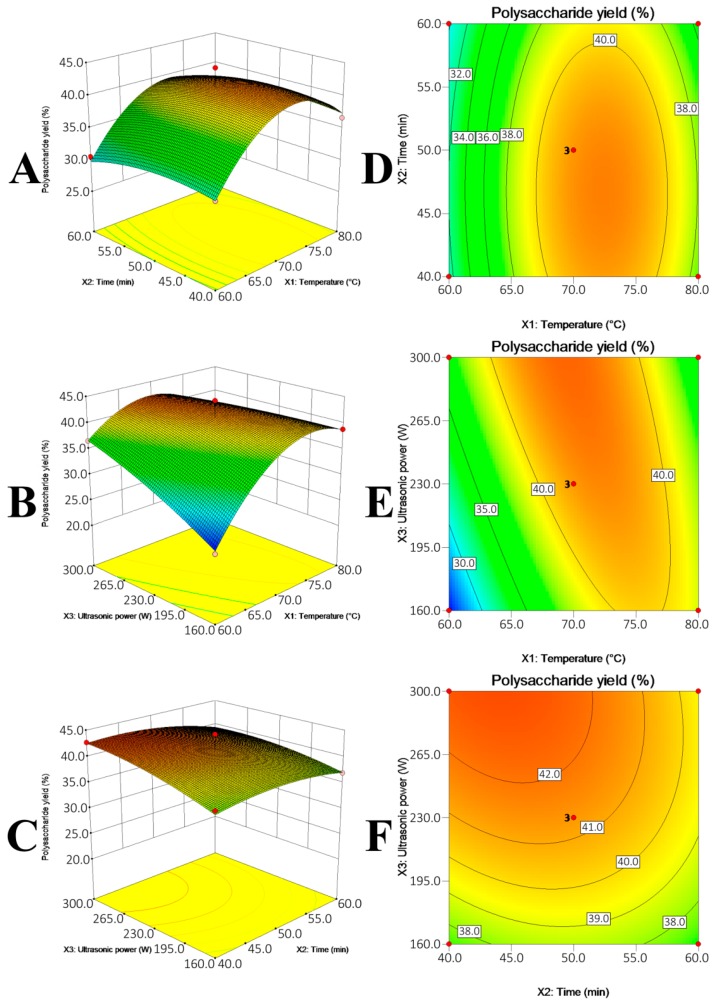
Response surface plot (**A**–**C**) and contour plots (**D**–**F**) showing the effects of temperature (*X*_1_), extraction time (*X*_2_) and ultrasonic power (*X*_3_) on the polysaccharide yield (*Y*) of DO.

The RSM with BBD actually not only generates optimal UAE conditions, but also comprehensively indicates how the UAE conditions differently affected the polysaccharide recovery. The effects of multiple UAE conditions, i.e., extraction temperature (*X*_1_), extraction time (*X*_2_) and ultrasonic power (*X*_3_), on the polysaccharide yields from GR, AR and DO were summarized in Figure 3, Figure 4 and Figure 5 and Table 2.

Three coefficients, namely the linear coefficient, cross product coefficient and quadratic term coefficient differently correlated the variables (UAE conditions) with the polysaccharide yields. To be specific, the linear coefficient and cross product coefficient indicated the linear effects (i.e., linear increase) of single variable (i.e., *X*_1_, *X*_2_ or *X*_3_) and effects of interaction of double variables (e.g., *X*_1_*X*_2_) on the polysaccharides yields, respectively, while the quadratic term coefficient represented that the single variable exhibited quadratic effects on the polysaccharides yields. Their *p*-values expressed the effects significantly (*p* < 0.05) or insignificantly (*p* > 0.05). As listed in Table 2, the single variables that significantly affected the polysaccharide yields from GR, AR and DO were different, i.e., extraction temperature (*X*_1_) for GR (*p* = 0.0142); extraction temperature (*X*_1_) (*p* < 0.0001) and time (*X*_2_) (*p* = 0.0049) for AR; extraction temperature (*X*_1_) for DO (*p* = 0.0071) were the significant coefficients. Besides, the interaction between extraction temperature (*X*_1_) and ultrasonic power (*X*_3_) for DO (Figure 5E) was significant to the polysaccharide yield (*p_X_*_1*X*3_ = 0.0095). The *p*-value of quadratic term coefficient *X*_1_^2^ for three models (*p* < 0.0001 for GR; *p* = 0.0001 for AR; *p* = 0.0012 for DO) and ultrasonic power *X*_3_^2^ (*p* = 0.0179) for GR model were small, which suggested that extraction temperature (*X*_1_) for the three models and ultrasonic power (*X*_3_) for GR exhibited quadratic effects on the dependent response (Figure 3A,B, Figure 4A,B and Figure 5A,B). Taking GR as an example to illustrate the quadratic effects by multiple variables, the polysaccharide yield increased with temperature from 70 to 80 °C and decreased afterward when ultrasonic power was fixed. Similarly, the polysaccharide yield increased with the increase in ultrasonic power from 160 W to 265 W and declined with the continued promotion of ultrasonic power from 265 W to 300 W (Figure 3B). In short, the multiple UAE conditions could significantly affect the polysaccharide yields, and that optimum conditions for different polysaccharides were different.

### 2.5. Effects of Structural Properties on Polysaccharide Recovery by UAE

**Figure 6:**
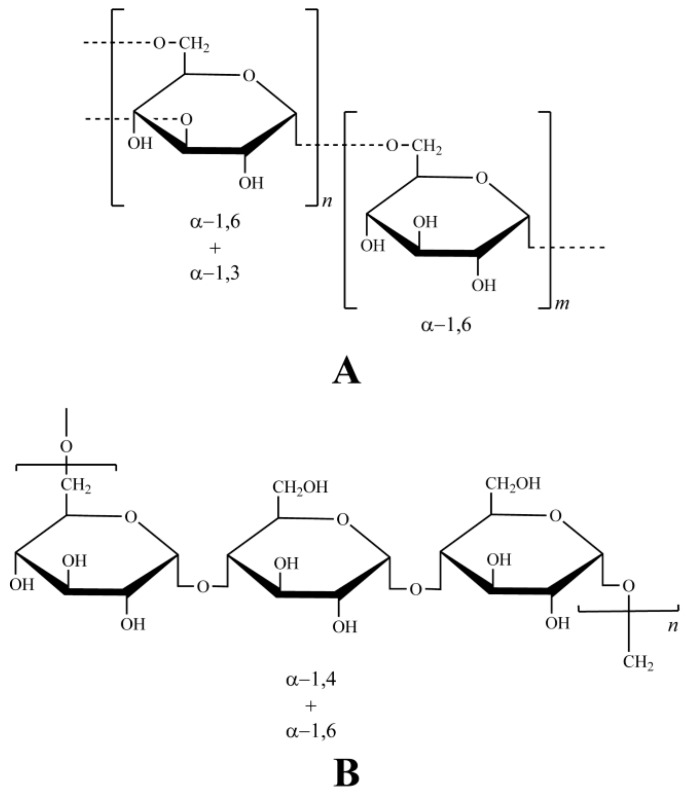
Chemical structures of standard glucans, branched dextran (**A**) and unbranched pullulan (**B**).

To further clearly observe how structural properties of polysaccharides contribute differently to their extraction recovery by UAE and BWE, we selected two standard glucans, namely dextrans and pullulans, for spike recovery testing. Dextrans are a type of branched glucans, consisting of straight chain linked by α-1,6-glycosidic bonds and branched by α-1,3 linkages, while pullulans are unbranched glucans composed by linear maltotriose units(α-1,4-glycosidic bonds linked trisaccharide) that are connected with α-1,6 linkages (Figure 6).

Obviously the series of dextrans and pullulans possessed different structural properties in terms of degree of polymerization as well as the types of sugar chain and glucosidic linkage. The AR material was employed as the spiked sample, in which the original AR carbohydrates were removed by BWE to exclude detection interference. The optimal UAE and BWE conditions for AR polysaccharides were used for the extraction of spiked samples. Then the recovered dextrans and pullulans were analyzed by HPGPC-CAD, and the quantitation was performed by the established standard curves (Table 4).

**Figure 7:**
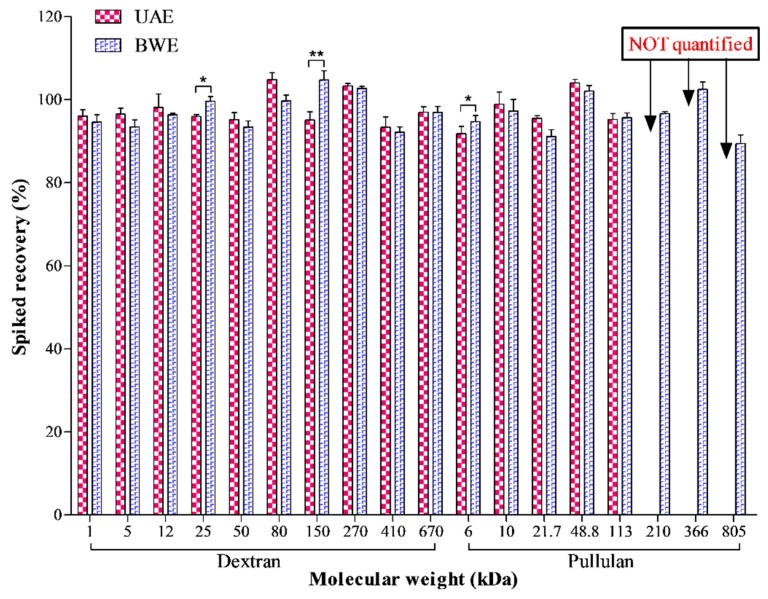
Quantitation results of recovered standard glucans by UAE and BWE * *p* < 0.05; ** *p* < 0.01.

**Figure 8:**
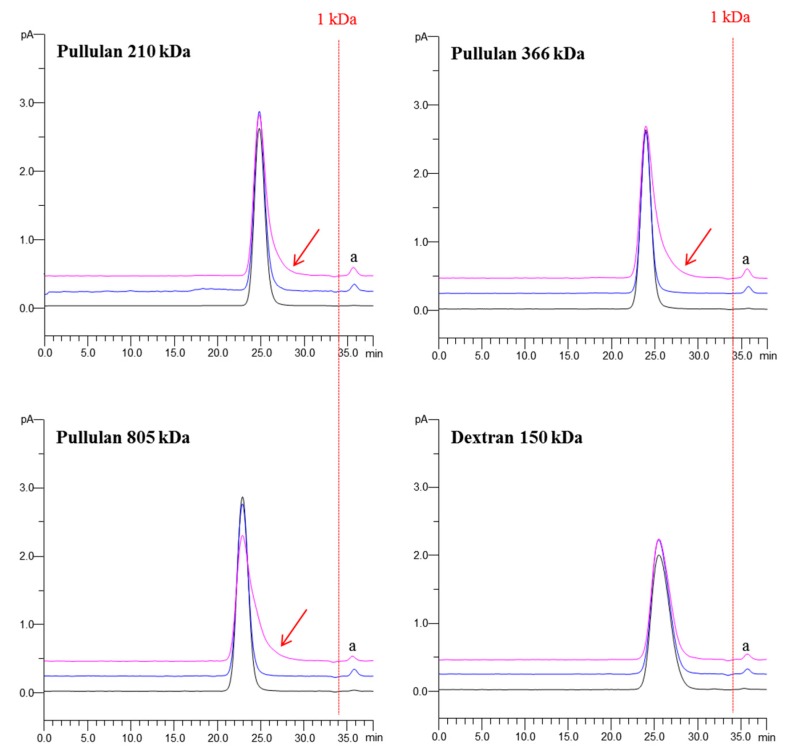
HPGPC-CAD chromatograms of typical recovered standard glucans by UAE and BWE — untreated; –– UAE; –– BWE.

The peak symmetry and width of the recovered glucans were regarded as measurements to determine whether the glucans could be quantified or not due to the substantial qualitative change caused by the extraction, the glucans whose peak symmetry factor or width varied over 5% by UAE were not quantified. The spike recovery results of dextrans and pullulans are displayed in Figure 7, and typical chromatograms are shown in Figure 8. 

As seen in the figures, the glucans responded differently to the extractions. Most obviously, three pullulans with higher molecular weights (210 kDa, 366 kDa, 805 kDa) were severely degraded by UAE, as evidenced by significantly tailed peaks (noted by red arrows, Figure 8A–C), i.e., elevated symmetry factor (210 kDa: from 1.16 to 1.63; 366 kDa: from 1.07 to 1.88; 805 kDa: from 1.08 to 1.97) and broadened peak width (210 kDa: from 1.35 to 1.58; 366 kDa: from 1.34 to 1.74; 805 kDa: from 1.46 to 2.09). Moreover, the degradation appeared to largely happen at the center of the chains, given the narrow molecular weight variation, but a new peak **a** with molecular weight less than 1 kDa was found in the recovered products of the three pullulans. Considering its low molecular weight, we speculated that the peak **a** should be derived from minor terminal degradation. Differently, 91.12 %–104.74% of all spiked dextrans and pullulans were recovered by BWE, while UAE also retrieved 91.75 %–104.83% all dextrans and the remaining pullulans with lower molecular weights (6 kDa–113 kDa) (Figure 7). The high recovery rates advised that the impacts of UAE and BWE on these glucans were mild, regardless of their structural differences. The new peak **a** was also detected in the chromatograms of these recovered glucans (Figure 8). This suggests formation of terminal-degraded products from the glucans induced by ultrasonic or thermal treatment [41]. Besides, significantly deceased (*p* < 0.05 or *p* < 0.01) rates of recovery by UAE were also determined on several glucans (25 kDa and 150 kDa dextrans, 6 kDa pullulan) (Figure 7), suggesting additional degradations by UAE over BWE. To sum up, the spike recovery testing on pullulan further evidenced that polysaccharides with higher molecular weight are indeed easier to be degraded by UAE. In addition, unbranched polysaccharides should be more susceptible by UAE than branched polysaccharides given the less recovered pullulans. Furthermore, α-1,4-glycosidic bonds might be more fragile than α-1,6-glycosidic bonds in the pullulans since the latter in the straight chains of dextrans were quite resistant to UAE, but this requires further experimental verification.

## 3. Materials and Methods

### 3.1. Chemicals, Reagents and Herbal Materials

Ammonium acetate was (purity ≥ 98%, chromatographic grade) purchased from Sigma-Aldrich (St. Louis, MO, USA). Deionized water was prepared by Millipore Milli Q-Plus system (Bedford, MA, USA). 98% sulfuric acid from RCI Labscan (Bangkok, Thailand), ethanol (analytical grade) from Merck (Darmstadt, Germany), and phenol from Sigma-Aldrich were used. The dextran and pullulan reference substances (Figure 6) with known molecular sizes (1–670 kDa for dextrans, 6–805 kDa for pullulans), and d-glucose were purchased from Sigma-Aldrich and Shodex (Tokyo, Japan). The herbal materials of GR, AR and DO were purchased from their geo-authentic product areas, i.e., the Jilin, Inner Mongilia and Anhui Province of PRC, respectively, and authenticated by Prof. Hu-Biao Chen. Voucher specimens of these samples were deposited at the School of Chinese Medicine, Hong Kong Baptist University, Kowloon Tong, Hong Kong.

### 3.2. Sample Preparation

#### 3.2.1. UAE

##### Box-Behnken Design and Statistical Analysis

The UAE conditions were examined by RSM with a Box-Behnken design (BBD). Based on the results of single-factor tests of each herbal medicine (Figure S1), the level ranges of extraction temperature (*X*_1_), extraction time (*X*_2_) and ultrasonic power (*X*_3_) for extraction of GR, AR and DO were determined.

With the three selected independent variables, polysaccharide yield (*Y*) was taken as the dependent response of the designed experiments. The coded levels and actual values of the independent variables were shown in Table 5, for which the actual levels of the coded variables were determined according to Equation (4).

(4) $$x_{i}=\frac{X_{i}-X_{0}}{∆X}$$

In Equation (4), $x_{i}$ refers to the coded level while $X_{i}$ is the actual value of the variable, $X_{0}$ represents the actual value of the variable at centre point and ∆X is the step change value. Fifteen experimental runs in triplicate, with three center points, were randomized to minimize the effects of unexpected variables in the observed responses. The software Design-Expert 8.0 (Stat-Ease, Inc., Minneapolis, MN, USA) was applied for generating experimental design, regression model (Equation (5)) and statistical analysis.

(5) $$Y=\beta_{0}+\overset{3}{\underset{i=1}{\sum }}\beta_{i}X_{i}+\overset{3}{\underset{i=1}{\sum }}\beta_{ii}X_{i}^{2}+\overset{3}{\underset{i=1}{\sum }}\overset{3}{\underset{j=i+1}{\sum }}\beta_{ij}X_{i}X_{j}$$

In Equation (5), *Y* refers to the dependent variable, which is the extraction yield of crude polysaccharides in herbal medicines. *β*_0_ is a constant while *β_i_*, *β_ii_* and *β_ij_* are regression coefficients estimated by the model for linearity, square and interaction. *X_i_* and *X_j_* are the different levels of independent variables. With the regression model deduced, three additional confirmation experiments were conducted to verify the validity of the regression model.

##### Extraction Procedure

Herbal material was oven-dried and powdered by a RT-04 grinder (Rong Tsong Precision Technology Co., Taichung, Taiwan) to pass through 80-mesh sieve. Ten mL deionized distilled water was added to 0.05 g of GR and AR powder and 0.1 g of DO powder in a flat-bottomed 20-mL sample vial with 20 mm diameter. The extraction was performed with a CP2600D ultrasonic machine (Crest, Trenton, NJ, USA) under water bath working at a frequency of 45 kHz with a usable capacity of 26 L. The sample vials was placed in the middle of the water bath with 11 cm above the transducer and the water level was kept at approximately 2.5 cm from the top. The extraction temperature, time and ultrasonic power were set according to the corresponding experimental design (Table 1). The extract of each run in triplicate was centrifuged and the supernatants were then obtained for further analysis.

#### 3.2.2. BWE

For each sample, 0.1 g of powder was extracted with deionized distilled water at 100 °C (10 mL × 1 h × 2 times). The extracted solutions were combined and then centrifuged at 4000 rpm for 10 min. The supernatants were transferred for subsequent analysis.

#### 3.2.3. Preparation of Crude Polysaccharide

The extraction solutions obtained by UAE and BWE for each sample were adjusted to same ratio of material (g) to solution (mL) (1:200 for GR and AR, 1:100 for DO). Two mL of the supernatants from UAE and BWE were then precipitated respectively with the addition of absolute ethanol to a final concentration of 90% (*v*/*v*) and incubated for 12 h in a 4 °C refrigerator. After centrifugation (4000 rpm for 10 min), the precipitates were collected, washed with ethanol, air-dried (water bath, 60 °C) to remove any residual ethanol, and then was completely re-dissolved in hot water (60 °C) (16 mL for GR and DO, 4 mL for AR) by drastic mechanical vibration for 2 h. Finally, the resulting solution was subjected to phenol-sulphuric acid analysis for quantitative determination of polysaccharides. For qualitative investigation, each solution was filtered through a 0.22 μm CA syringe filter (Agilent Technologies, Waltham, MA, USA) for HPGPC-CAD analysis.

### 3.3. Phenol-Sulphuric Acid Analysis

The amount of polysaccharide in the crude polysaccharide solutions was determined using phenol-sulphuric acid analysis by V-350 UV/VIS spectrophotometer from Jasco (Tokyo, Japan). 0.4 mL of each solution was mixed with 0.4 mL of 5% phenol and 2 mL concentrated sulphuric acid (98%). Using d-glucose as a reference standard to construct a standard curve under the same analytical conditions, the extraction yield of crude polysaccharide was calculated as follows:

(6) $$\mathrm{Polysaccharide}\;\mathrm{yield}\;(\%)\;=\;\frac{\mathrm{weight}\;\mathrm{of}\;\mathrm{determined}\;\mathrm{polysaccharide}\;(g)}{\mathrm{weight}\;\mathrm{of}\;\mathrm{sample}\;(g)}\;\times \;100\%$$

### 3.4. HPGPC-CAD analysis

The crude polysaccharide extracts of samples were qualitatively analyzed using HPGPC performed on a Dionex UltiMate 3000 series ultra-high performance liquid chromatography and diode array detector (UHPLC-DAD) system coupled with Dionex Corona Veo CAD from Thermo Scientific (Waltham, MA, USA). Two tandem TSK GMPW_XL_ columns (300 × 7.8 mm i.d., 10 μm) were employed for analysis. Ammonium acetate aqueous solution (20 mM) was used as mobile phase at a flow rate of 0.6 mL/min. The column temperature was constantly kept at 40 °C. The parameters of CAD were set as follows: data collection rate at 2 Hz, filter at 10 s, gain at 100 pA, nebulizer heater at 60 °C and gas regulator mode at analytical. UV detection wavelengths were set at 260 and 280 nm. An aliquot of 20 μL solution was injected for analysis.

Aqueous stock solutions of dextrans (2 mg/mL) with different molecular weights (1, 5, 12, 25, 50, 80, 150, 270, 410, and 670 kDa) and pullulans (2 mg/mL) (Figure 6) with different molecular weights (6, 10, 21.7, 48.8, 113, 210, 366 and 805 kDa) were injected into the HPGPC-CAD using the abovementioned conditions for the construction of the molecular weight-retention time calibration curve by plotting logarithm of the molecular weight versus retention time of each analyte.

### 3.5. Spiked Recovery Testing on Standard Glucans

The ten dextrans and eight pullulans mentioned above were accurately weighed (about 1 mg each) and then were respectively spiked into the blank powder of AR, which was prepared by repeated extraction until no sugar was detected by both phenol-sulphuric acid analysis and HPGPC-CAD analysis. The spiked samples were extracted in triplicate by the optimized UAE and BWE methods for AR, respectively. Subsequently the supernatant of each extract after centrifugation (4000 rpm, 10 min) was directly injected into HPGPC-CAD for qualitative and quantitative analysis.

Aqueous stock solutions of the standard glucans were diluted to appropriate concentrations to construct standard curves for the quantitative determination of recovered dextrans and pullulans. Five concentrations of each standard solution were analyzed by HPGPC-CAD, and then the calibration curves were established by plotting the logarithm of peak areas versus the logarithm of concentrations of each analyte.

## 4. Conclusions

In this study, using GR, AR and DO as illustrative samples, we experimentally confirmed that polysaccharides from herbal medicines by UAE were quantitatively and qualitatively different from those obtained by BWE. The powerful extraction ability and polysaccharide degradation caused by ultrasound collectively contributed to the differences. Furthermore, it was demonstrated that not only the UAE parameters (extraction temperature, time and ultrasonic power) but also the structural properties of the polysaccharides (polymerization degree, the types of sugar chain and glucosidic linkage) could substantially and differently affect the extraction ability and polysaccharide degradation, and thereby the extraction recovery of polysaccharides from herbal medicines by UAE. Because UAE-obtained polysaccharides are inconsistent with those obtained by the conventional preparation method (BWE) of herbal medicines used in clinical practice, we highly recommended that quantitative and qualitative effects of UAE on specific polysaccharides from herbal medicines, which could be decisive to their bioactivities, should be considered before employing it in any relevant chemical and pharmacological analysis.

## Figures and Tables

**Table 1:** Experimental designs of BBD and response values for polysaccharide yields (*n* = 3).

Run	*X*_1_	*X*_2_	*X*_3_	*Y*		
	Level of Temperature	Level of Extraction Time	Level of Ultrasonic Power	Polysaccharide Yield (%)		
				GR	AR	DO
**1**	1	0	−1	28.60	4.51	38.83
**2**	0	0	0	46.26	3.89	41.03
**3**	0	1	−1	43.56	4.04	36.93
**4**	−1	0	1	31.67	0.72	36.61
**5**	0	0	0	44.28	3.93	44.21
**6**	0	1	1	42.05	4.80	38.75
**7**	0	0	0	48.84	4.27	38.76
**8**	1	1	0	29.89	5.33	35.63
**9**	−1	1	0	37.17	0.90	30.55
**10**	−1	0	−1	33.77	0.61	24.63
**11**	1	−1	0	31.42	3.75	36.64
**12**	−1	−1	0	35.89	0.53	31.41
**13**	0	−1	−1	38.47	3.40	37.97
**14**	0	−1	1	45.22	3.86	42.69
**15**	1	0	1	27.04	4.24	35.45

**Table 2:** Analysis of variance for the fitted quadratic polynomial models of polysaccharide yields.

Source	Sum of Squares	Degree of Freedom	Mean Square	*F*-Value	*p*-Value
GR					
Model	681.96	9	75.77	17.73	0.0028
*X*_1_	58.02	1	58.02	13.58	0.0142
*X*_2_	0.34	1	0.34	0.081	0.7879
*X*_3_	0.32	1	0.32	0.074	0.7960
*X*_1_*X*_2_	1.98	1	1.98	0.46	0.5266
*X*_1_*X*_3_	0.074	1	0.074	0.017	0.9002
*X*_2_*X*_3_	17.05	1	17.05	3.99	0.1023
*X*_1_^2^	573.59	1	573.59	134.21	<0.0001
*X*_2_^2^	0.61	1	0.61	0.14	0.7202
*X*_3_^2^	51.31	1	51.31	12.01	0.0179
Residual	21.37	5	4.27		
Lack of Fit	10.95	3	3.65	0.70	0.6330
Pure Error	10.41	2	5.21		
Cor Total	703.32	14			
	R^2^ = 0.9696	Adj R^2^ = 0.9149	Pred R^2^ = 0.7175	C.V. = 5.50%	
AR					
Model	38.47	9	4.27	62.77	0.0001
*X*_1_	28.42	1	28.42	417.28	< 0.0001
*X*_2_	1.56	1	1.56	22.92	0.0049
*X*_3_	0.14	1	0.14	2.08	0.2084
*X*_1_*X*_2_	0.37	1	0.37	5.42	0.0673
*X*_1_*X*_3_	0.036	1	0.036	0.53	0.5004
*X*_2_*X*_3_	0.022	1	0.022	0.33	0.5913
*X*_1_^2^	7.82	1	7.82	114.81	0.0001
*X*_2_^2^	9.275 × 10^−3^	1	9.275 × 10^−3^	0.14	0.7272
*X*_3_^2^	0.012	1	0.012	0.18	0.6911
Residual	0.34	5	0.068		
Lack of Fit	0.25	3	0.085	1.97	0.3538
Pure Error	0.086	2	0.043		
Cor Total	38.81	14			
	R^2^ = 0.9912	Adj R^2^ = 0.9754	Pred R^2^ = 0.8901	C.V. = 8.03%	
DO					
Model	322.24	9	35.80	10.13	0.0101
*X*_1_	68.17	1	68.17	19.29	0.0071
*X*_2_	5.85	1	5.85	1.66	0.2545
*X*_3_	28.64	1	28.64	8.10	0.0360
*X*_1_*X*_2_	5.658 × 10^−3^	1	5.658 × 10^−3^	1.601 × 10^−3^	0.9696
*X*_1_*X*_3_	58.94	1	58.94	16.68	0.0095
*X*_2_*X*_3_	2.10	1	2.10	0.59	0.4761
*X*_1_^2^	155.59	1	155.59	44.02	0.0012
*X*_2_^2^	6.13	1	6.13	1.73	0.2451
*X*_3_^2^	3.44	1	3.44	0.97	0.3694
Residual	17.67	5	3.53		
Lack of Fit	2.69	3	0.90	0.12	0.9407
Pure Error	14.99	2	7.49		
Cor Total	339.91	14			
	R^2^ = 0.9480	Adj R^2^ = 0.8544	Pred R^2^ = 0.7743	C.V. = 5.13%	

**Table 3:** Verification results of the predicted optimal extraction condition for polysaccharides (*n* = 3).

Natural Samples	Condition	Temperature (°C)	Extraction Time (min)	Ultrasonic Power (W)	Polysaccharide Yield (%)
GR	Predicted	78.62	95.99	212.26	46.73
	Modified	80	96	230	47.95 ± 0.30
AR	Predicted	82.19	72	300	5.43
	Modified	80	72	300	5.24 ± 0.06
DO	Predicted	69.31	43.89	300	42.78
	Modified	70	44	300	42.37 ± 0.83

**Table 4:** Calibration curves of glucan standards for quantitation in spike recovery testing.

Glucan standard	Molecular Weight (kDa)	Equation	R^2^
Dextran	1	*y* = 0.8386*x* − 1.4103	0.9993
	5	*y* = 0.7752*x* − 1.1793	0.9994
	12	*y* = 0.8546*x* − 1.3745	0.9995
	25	*y* = 0.8748*x* − 1.4518	0.9997
	50	*y* = 0.8496*x* − 1.4220	0.9990
	80	*y* = 0.8648*x* − 1.4031	0.9999
	150	*y* = 0.8727*x* − 1.3954	0.9998
	270	*y* = 0.8829*x* − 1.6447	0.9992
	410	*y* = 0.9081*x* − 1.6390	0.9997
	670	*y* = 0.8660*x* − 1.4596	0.9990
Pullulan	6	*y* = 0.7573*x* − 1.2547	0.9998
	10	*y* = 0.8543*x* − 1.4567	0.9990
	21.7	*y* = 0.8066*x* − 1.4053	0.9994
	48.8	*y* = 0.9954*x* − 1.9075	0.9994
	113	*y* = 0.9061*x* − 1.7526	0.9994
	210	*y* = 0.9544*x* − 1.6836	0.9991
	366	*y* = 0.8757*x* − 1.5806	0.9991
	805	*y* = 0.7647*x* − 1.5348	0.9991

**Table 5:** Coded levels and actual values of independent factors for BBD experiments.

Natural Samples	Independent Variables	Level		
		−1	0	+1
**GR**	Temperature (*X*_1_) (°C)	70	80	90
	Extraction time (*X*_2_) (min)	48	72	96
	Ultrasonic power (*X*_3_) (W)	160	230	300
**AR**	Temperature (*X*_1_) (°C)	65	75	85
	Extraction time (*X*_2_) (min)	24	48	72
	Ultrasonic power (*X*_3_) (W)	160	230	300
**DO**	Temperature (*X*_1_) (°C)	60	70	80
	Extraction time (*X*_2_) (min)	40	50	60
	Ultrasonic power (*X*_3_) (W)	160	230	300

## Supplementary Materials

Supplementary materials can be accessed at: http://www.mdpi.com/1420-3049/21/11/1569/s1.
