# Supplementary material for: Ultrasound-Assisted Extraction May Not Be a Better Alternative Approach than Conventional Boiling for Extracting Polysaccharides from Herbal Medicines
Source: Molecules. 2016 Nov 18;21(11):1569. doi: 10.3390/molecules21111569 (PMC6274063; doi:10.3390/molecules21111569)
Supplement: Supplementary file 1 [file molecules-21-01569-s001.pdf]

Supplementary Materials: Ultrasound-Assisted Extraction May Not Be a Better Alternative Approach than Conventional Boiling for Extracting Polysaccharides from Herbal Medicines

Ka-Man Yip, Jun Xu, Wing-Sum Tong, Shan-Shan Zhou, Tao Yi, Zhong-Zhen Zhao and Hu-Biao Chen

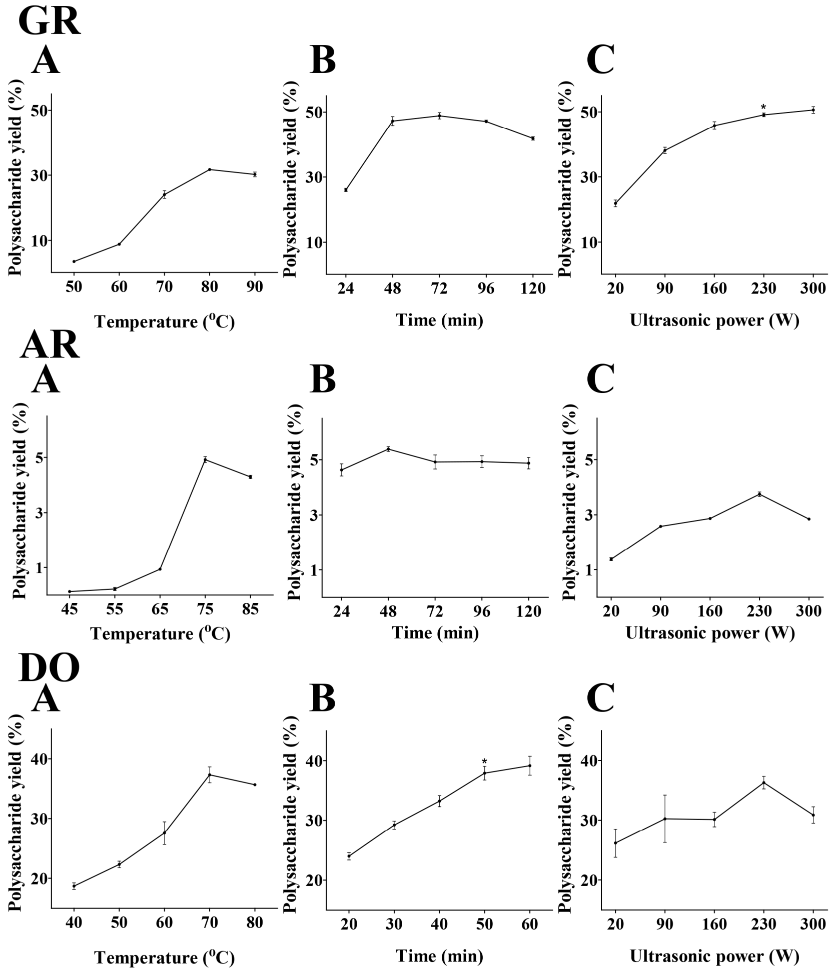

Figure S1. Effect of different temperature (**A**); extraction time (**B**) and ultrasonic power (**C**) on the polysaccharide yield in the herbal samples by single-factor test.* $p < 0.05$, compared with the previous one.
